# Raman spectroscopy uncovers biochemical tissue-related features of extracellular vesicles from mesenchymal stromal cells

**DOI:** 10.1038/s41598-017-10448-1

**Published:** 2017-08-29

**Authors:** Alice Gualerzi, Stefania Niada, Chiara Giannasi, Silvia Picciolini, Carlo Morasso, Renzo Vanna, Valeria Rossella, Massimo Masserini, Marzia Bedoni, Fabio Ciceri, Maria Ester Bernardo, Anna Teresa Brini, Furio Gramatica

**Affiliations:** 1 Laboratory of Nanomedicine and Clinical Biophotonics, IRCCS Fondazione Don Carlo Gnocchi ONLUS, Milano, Italy; 2 Dipartimento di Scienze Biomediche, Chirurgiche ed Odontoiatriche, Università degli Studi di Milano, Milano, Italy; 3 Laboratorio di Applicazioni Biotecnologiche, IRCCS Istituto Ortopedico Galeazzi, Milano, Italy; 4 Nanomedicine Center NANOMIB, School of Medicine and Surgery, University of Milano-Bicocca, Monza, Italy; 5 San Raffaele Telethon Institute for Gene Therapy (SR-TIGET), Pediatric Immunohematology, San Raffaele Scientific Institute, Milano, Italy; 6 Hematology and Bone Marrow Transplantation Unit, San Raffaele Scientific Institute, Milano, Italy

## Abstract

Extracellular vesicles (EVs) from mesenchymal stromal cells (MSC) are emerging as valuable therapeutic agents for tissue regeneration and immunomodulation, but their clinical applications have so far been limited by the technical restraints of current isolation and characterisation procedures. This study shows for the first time the successful application of Raman spectroscopy as label-free, sensitive and reproducible means of carrying out the routine bulk characterisation of MSC-derived vesicles before their use *in vitro* or *in vivo*, thus promoting the translation of EV research to clinical practice. The Raman spectra of the EVs of bone marrow and adipose tissue-derived MSCs were compared with human dermal fibroblast EVs in order to demonstrate the ability of the method to distinguish the vesicles of the three cytotypes automatically with an accuracy of 93.7%. Our data attribute a Raman fingerprint to EVs from undifferentiated and differentiated cells of diverse tissue origin, and provide insights into the biochemical characteristics of EVs from different sources and into the differential contribution of sphingomyelin, gangliosides and phosphatidilcholine to the Raman spectra themselves.

## Introduction

Extracellular vesicles (EVs) are a heterogeneous group of membrane-bound vesicles that are constitutively released by cells of different tissue origins. Past controversies concerning nomenclature have now been resolved by the scientific community, which defines EVs as the group of particles made up of exosomes, microvesicles and apoptotic bodies^1^. Exosomes (30–100 nm) and microvesicles (up to 1000 nm) differ in size and cellular origin, but both mediate intercellular communication within a tissue and among organs thanks to body fluid transportation^1^.

As is the case for most body cells, part of the secretome of mesenchymal stromal cells (MSCs) includes exosomes and microvesicles, which are currently being investigated because of their striking regenerative and immunomodulating potential. The bioactive molecules loaded onto/into EVs are involved in the paracrine effects of stem cells, and even the membrane constituents of vesicles seem to trigger intracellular protective/regenerative pathways in recipient cells^2^. It has been suggested that MSC-derived EVs may be sometimes even more therapeutically valuable than whole cells, because of their remarkable handling advantages, which can accelerate their clinical application in the so-called *cell therapy without cells* ^3^. The possibility of overcoming the cell therapy drawbacks of having to administer living, replicating and difficult to control cells is currently one of the main challenges facing regenerative medicine, and EVs can be an effective means of stimulating the restoration of organ function through tissue regeneration and repair in the context of an integrated strategy of *regenerative rehabilitation* ^4^.

Over the last ten years, many studies have demonstrated the role that MSC-derived EVs can play in tissue repair and immunomodulation^5, 6^ and, in 2014, EVs ability to influence the activity of recipient cells and regulate immune responses was successfully exploited in a patient undergoing allogeneic hematopoietic stem cell transplantation who developed therapy-refractory graft-versus-host disease^7^. Their regenerative potential has also been assessed in *in vitro* and *in vivo* models of many diseases affecting heart^8–10^, kidney^11–13^, liver^14, 15^, bone and cartilage^16, 17^, muscle^18^, skin^19^, and central nervous system^20–23^.

However, there are still concerns about the effect that the source of MSCs and cell culture conditions can have on EV production and characteristics as there is no standardised and optimised method for isolating and characterising EVs. Furthermore, the technical restraints of current techniques have limited their potential use in regenerative medicine^24, 25^ by preventing reproducible quality and safety assessments^26^.

The aim of this study was to test Raman spectroscopy (RS) as a label-free, non-destructive, sensitive, rapid and automatable means of carrying out the bulk characterization of EVs. This technique provides a spectrum that qualitatively and quantitatively describes the chemical composition of a sample and thus avoids the need for specific protein biomarkers. It has been widely used in the pharmaceutical industry as a mean of verifying raw materials and quality controlling drug production, and we suggest it could help in purity and quality checking vesicle suspensions. It has already proved its value by characterising a wide range of cells and tissue samples for the purposes of basic research, and as an innovative alternative to classic, time-consuming and operator-dependent diagnostic methods^27–34^. In the field of regenerative medicine, it has been used to analyse undifferentiated and differentiated human and murine embryonic stem cells^35–37^ and to monitor MSCs stimulated towards osteogenic differentiation^38, 39^. Efforts have also been made to develop Raman-based methods for the individual characterisation of human vesicles^40, 41^, but although these have provided information at single-vesicle level, they are still far from being used diagnostically. What is required to allow the immediate transferability of EV research to clinical practice is a procedure that allows i) the rapid characterisation of a sample before its use *in vitro* or *in vivo*; ii) the identification of fingerprints of the EV populations used for regenerative purposes in order to determine the best experimental settings and compare results from different cell sources; and iii) the routine application of the analysis. The third point should be favoured by the current availability of portable Raman spectrometers that can automatically scan and analyse complex samples, which could bring Raman analysis easily in the reach of most laboratories. RS is much more suitable for achieving these goals than the widely used techniques of immunoblotting, cytofluorimetry and spectrometry because it can provide reproducible results quickly and in a label-free manner, and only requires tiny sample volumes in comparison with the large amounts needed by other methods, which cannot easily cope with the nanoscalar dimensions of exosomes.

This study provides the first Raman-based characterisation of the EVs of human MSCs isolated from bone-marrow (bone marrow mesenchymal stromal cells, BM-MSCs) and subcutaneous adipose tissue (adipose tissue mesenchymal stromal cells, ASCs). The results were compared with those obtained using EVs released by dermal fibroblasts (DFs), in order to verify the ability of Raman analysis to distinguish vesicles from undifferentiated and differentiated cells, and gain insights into the biochemical features of EVs from different sources. Multivariate analysis was used to assess spectral differences and automatically distinguish the three groups. In addition, given the growing body of evidence concerning the pivotal role of lipids in mediating EV functions^42^, we also evaluated the contribution of lipid membrane constituents to the Raman spectra. Our findings provide evidence supporting the use of RS for the routine characterisation of MSC-derived EVs before their *in vitro/in vivo* application.

## Results

### EV characterisation

EVs were isolated from BM-MSCs, ASCs and DFs following a multi-step ultracentrifugation protocol^43^ and characterised by immunoblotting and transmission electron microscopy (TEM) to verify their peculiar features as suggested by the International Society for Extracellular Vesicles (ISEV)^44^.

**Figure 1:**
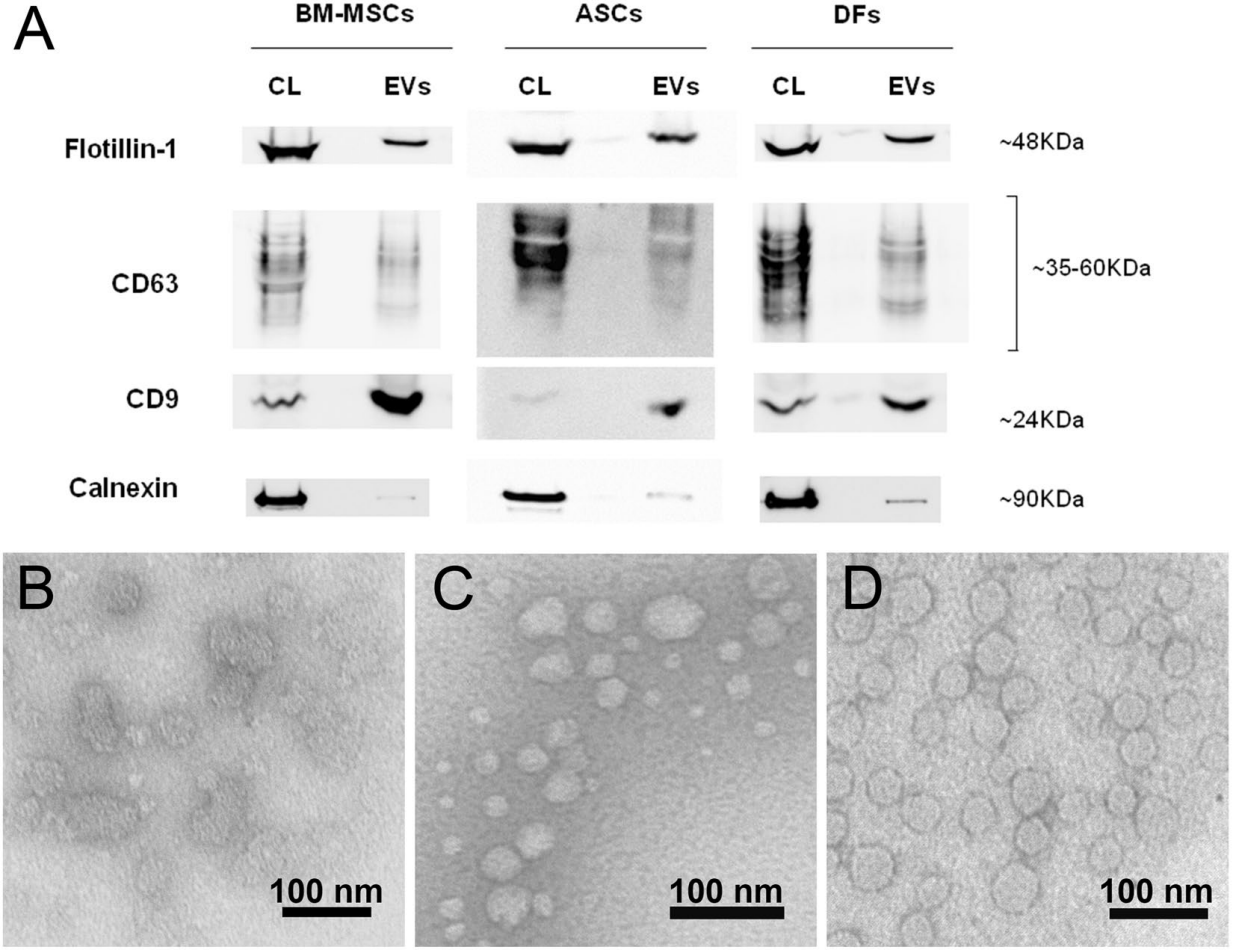
EV characterisation by means of Western blotting and transmission electron microscopy. (**A**) Western blot analysis of extracellular vesicles-enriched fractions (EVs) produced by BM-MSC, ASCs and DFs using the indicated antibodies. Flotillin-1, CD63 and CD9 are positive markers for EVs and Calnexin is considered a negative one. Corresponding cell lysate (CL) was used as control and depicts the specificity of the three antibodies. Western blots were cropped to improve clarity. All bands within the range of the molecular markers were retained and processing of the film was applied equally across the entire image. (**B**–**D**) Representative transmission electron photomicrographs of ultracentrifuged EVs from BM-MSCs (**B**), ASCs (**C**) and DFs (**D**) conditioned medium. The TEM images were used for size measurements. Bars = 100 nm.

Immunoblotting confirmed the presence of EVs carrying flotillin-1, CD63 and CD9, and a significant reduction in calnexin-positive vesicles (Fig. 1A). The TEM images (Fig. 1B–D) confirmed the typical morphology of the EVs, whose ultrastructure and size were consistent with published data^45^. The vesicles in all of the samples were round (Fig. 1B–D) and their general mean diameter as calculated on the TEM images was 46.5 nm (±15.8 nm) with slight differences among cell groups. Supplementary Figure S1 shows a box plot with all of the recorded measurements.

### Raman spectroscopy biochemical overview of Evs

Freshly isolated EVs were analy**s**ed by RS in the spectral ranges of 500–1800 cm^−1^ and 2600–3200 cm^−1^, the most significant regions of the Raman spectrum for biological specimens. The spectra were obtained from random spots of air-dried drops of EV suspension and, given the size of the laser beam, we speculate that every spectrum described the biochemical features of small clusters of aggregated EVs.

**Figure 2:**
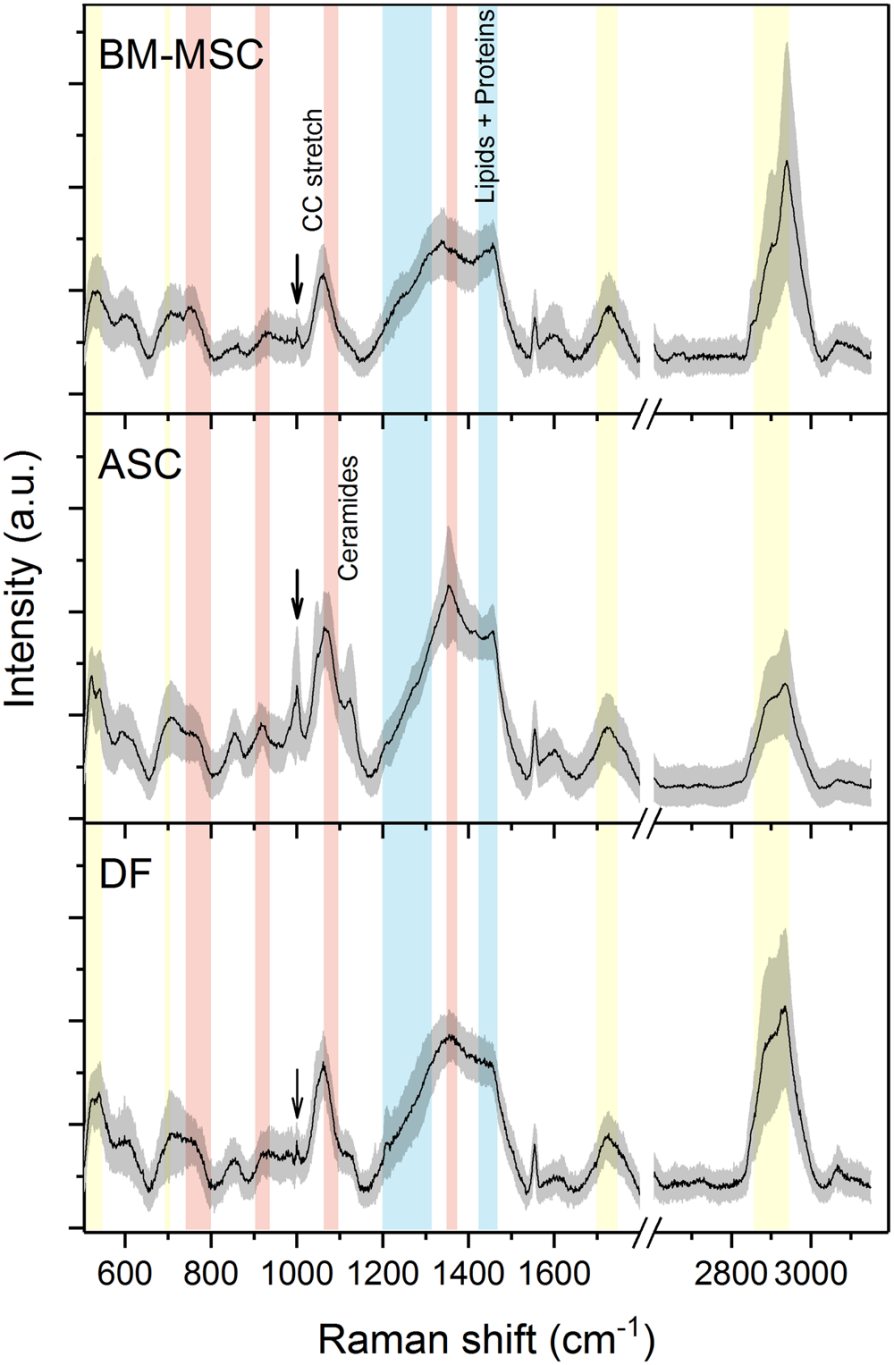
Raman fingerprint of BM-MSCs, ASCs, and DFs. Average Raman spectra obtained using an excitation wavelength of 532 nm and 10 seconds of exposure for 2 accumulations for each spectrum. The solid black line indicates the average of 40–50 spectra ± 1 standard deviation (shaded grey areas). The Raman bands corresponding to lipids are highlighted in yellow (500–540 cm^−1^; the band centred at 700 cm^−1^; 1700–1740 cm^−1^; 2850–2950 cm^−1^), those corresponding to proteins are in blue (1200–1300 cm^−1^; the band centred at 1450 cm^−1^), and those corresponding to nucleic acids are in red (720–820 cm^−1^; the band centred at 915 cm^−1^; 1060–1100 cm^−1^; the band centred at 1360 cm^−1^). The arrows indicate the 1003 cm^−1^ peak of Phenylalanine.

Figure 2 shows representative mean Raman spectra (±1 standard deviation) of the vesicles isolated from the supernatants of BM-MSCs, ASCs and DFs. Each mean spectrum represents the average of 40–50 independent recordings obtained from all of the donors of the same cell type. The overall homogeneity in the spectra from the same tissue source underlines the reproducibility of the analytical method, which is not affected by the intrinsic inter-individual variability of donors.

The spectra showed characteristic Raman bands of nucleic acids (NAs, 720–820 cm^−1^), phenylalanine (Phe, 1003 cm^−1^), lipid and protein markers such as CH and CH_2_ groups (bands respectively centred at 1450 cm^−1^ and 2940 cm^−1^) (Fig. 2 and Table 1). In particular, lipids made a large contribution, which is in line with previously reported spectroscopic evidence^40, 41, 46^. Lipid content was characterised by peaks attributable to cholesterol and cholesterol ester (537; 702; 1130; 1442 cm^−1^) and peaks of varying intensity corresponding to the C-C stretch (around 1100 cm^−1^) and CH, CH_2_, and CH_3_ bonds (in the spectral range 2600–3200 cm^−1^). In addition, the areas usually assigned to NA bases (718; 748; 782 cm^−1^) and phosphate backbone (785; ~1060 cm^−1^) were variably prominent in the three average spectra. This is in line with many data demonstrating that EVs contain intact mRNA, long non-coding RNA, miRNA and other forms of RNA loaded into EVs^42^. The recurrent peaks attributable to proline/hydroxyproline (853; 920; 1206 cm^−1^) and tryptophan (752–760; 1208; 1360; 1555 cm^−1^) may be related to differences in cell metabolism and responses to serum-deprived culture conditions. Proline is known to be a signalling molecule and a sensor of cellular energy status when responding to metabolic stress^47^, and the kynurenine pathway of tryptophan has been reported as being involved in the immunosuppressive effects of MSCs^48^.

**Table 1:** Main Raman peak assignments from literature refs 65–67.

Position (cm^−1^)	Component
520	Phosphatidyinositol
524	Phosphatidylserine
**537**	**Cholesterol ester**
540	Glucose-saccharide band
596	Phosphatidylinositol
701–703	Cholesterol ester
720–820	Nucleic acids
**752–760**	**Tryptophan**
840–860	Polysaccharide structure
941	Polysaccharide structure
**995**	**C-O band of ribose**
1003	Phenylalanine
1048	Glycogen
**1054**	**C-O and C-N stretching of proteins**
1060–1095	C-C vibrations of lipids and carbohydrates
	PO_2_^−^ stretching of nucleic acids
1120	C-O band of ribose; carotenoids
**1127**	**C-N stretching of proteins; ceramides**
1200–1300	Amide III
	*1230–1240 for β-sheets; 1260–1300 for α-helix*
**1298**	**Fatty acids**
**1336**	**CH3CH2 wagging mode of polynucleotide chain (purine bases)**
**1337**	**Tryptophan; Glycine backbone and Proline side chain**
1357; 1361	Guanine
1360	Tryptophan
1420–1480	CH functional groups of nucleic acids, proteins and lipids
**1520–38**	**Carotenoids**
1555–1558	Tryptophan
1716–1740	C=O group
2853–2881	CH_2_ symmetric and asymmetric stretches of lipids
**2910**	**CH**_**3**_ **stretching vibrations**
**2940**	**CH and CH**_**2**_ **in lipids and proteins**

The major divergent peaks are highlighted in bold characters.

Comparison of the average spectra revealed many differences between the cytotypes (highlighted in bold characters in Table 1), suggesting discrepancies in the panel of protein biomarkers and lipid content of vesicles, although it is difficult to attribute divergences in peak intensity to specific molecules. The presence of a 1127 cm^−1^ peak seemed to distinguish ASC spectra from those of both BM-MSC and DF EVs. The comparison of ASC and DF data highlighted minor divergences in the spectral range 2600–3200 cm^−1^, which is greatly influenced by lipid molecules thus indicating similarities in the lipid content of ASC- and DF-derived vesicles.

### Lipid membrane constituents account for spectral differences

**Figure 3:**
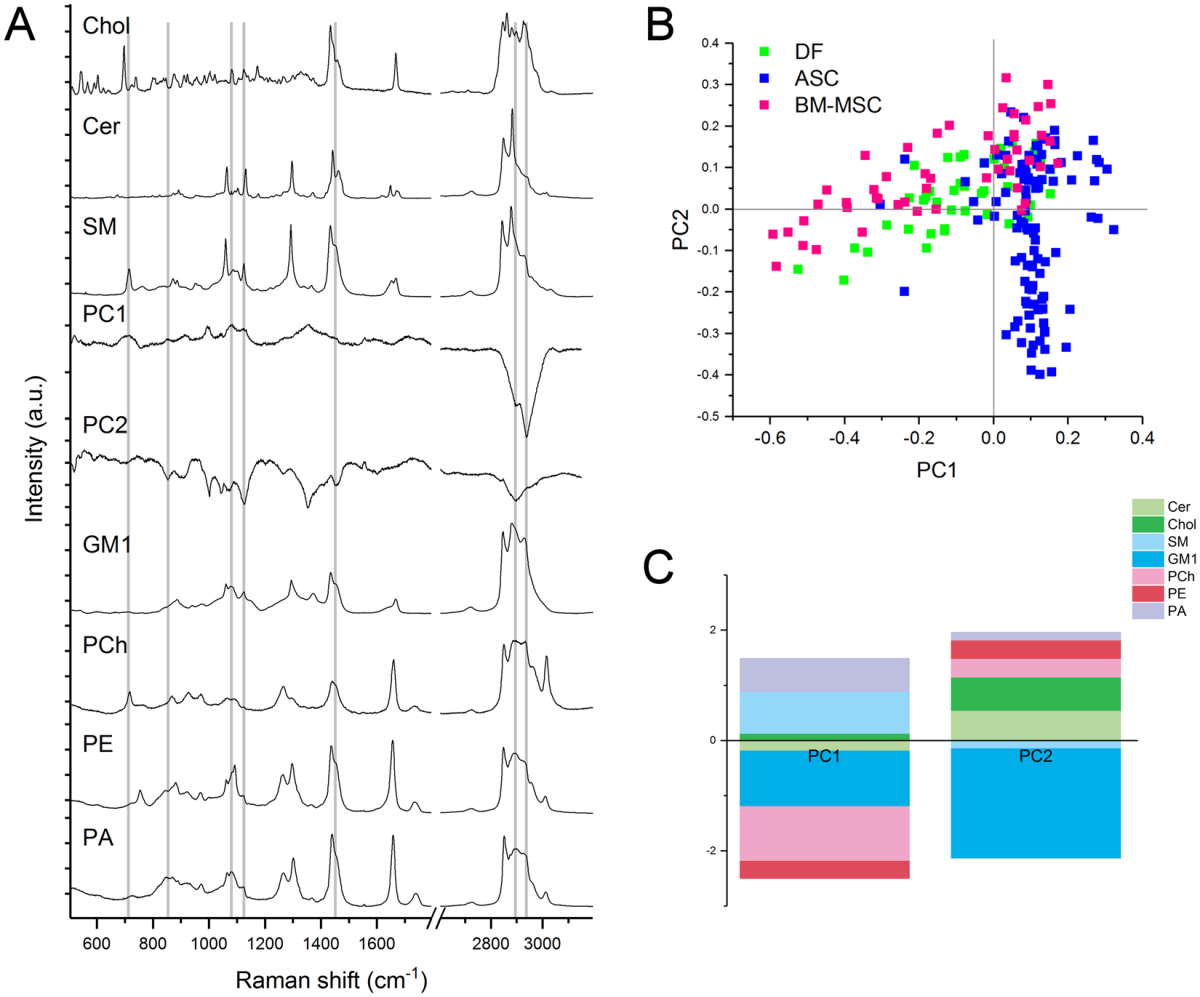
Multivariate analysis of the Raman spectra. (**A**) Raman spectra of reference lipid molecules, PC1 and PC2 that were considered for CLS fitting. The grey lines highlight the correspondences between the peaks of the standard lipids and the PC loadings. (**B**) Scatter plot of the PCA results showing the PC1 and PC2 scores assigned to each spectrum recorded from the EVs of BM-MSCs (pink), ASCs (blue) and DFs (green). Each square represents one spectrum. The scatter plot shows that a positive PC1 score described ASC-derived EVs better than those derived from DFs and BM-MSCs, whereas a positive PC2 score better described EVs derived from BM-MSC. (**C**) CLS scores obtained after fitting the spectra of Cer (light green), Chol (green), SM (light blue), GM1 (blue), PCh (pink), PE (red) and PA (violet) with the PC1 and PC2 loadings. The positive and negative scores obtained after CLS fitting are visualized as a bar graph and respectively indicate the contribution of each standard molecules to the positive or negative peaks visible in the PC1 and PC2 loadings. Cer: ceramide; Chol: cholesterol; SM: sphingomyelin; GM1: monosialotetrahexosylganglioside; PCh: phosphatidylcholine; PE: phosphatidylethanolamine; PA: phosphatidic acid.

Principal Component Analysis (PCA) was used to simplify the original data (n = 198) and all of the spectra were collectively represented by their principal components (PCs). Starting from PC1 (which accounted for 37.1% of total variance), the subsequent PCs describe differences in the Raman fingerprint that were progressively less prominent (Supplementary Table S1 and Supplementary Fig. S2). The first 2 PCs (Fig. 3A) were used to build the scatter plot shown in Fig. 3B. Combined analysis of the scatter plot and the PC1 and PC2 spectra revealed that the positive loadings in the PC1 spectrum mainly describe the biochemical features of ASC-derived EVs, positive peaks in PC2 represent BM-MSC vesicles rather than ASC or DF vesicles. One-way ANOVA performed on PC1 and PC2 scores demonstrated that the means of each group were significantly different (Prob > F < 0.05), despite within-group variance (Supplementary Table S2).

Based on the simple premise that a spectrum from a mixture of chemical ingredients is a mixture of the spectra from the pure ingredients, the PC1 and PC2 loadings were least squares fitted (classical least square (CLS) fitting) with specific reference spectra to investigate one possible cause of the observed spectral differences, following a previously reported procedure^40^. As we observed that the most variable spectral intervals in PC1 and PC2 were related to lipids, the membrane components cholesterol (Chol), ceramide (Cer), sphingomyelin (SM), phosphatidylcholine (PCh), phosphatidylethanolamine (PE), phosphatidic acid (PA), and monosialotetrahexosylganglioside (GM1, reference molecule for monosialoganglioside family) were used for CLS fitting. Lipid reference molecules were preferred to protein markers because proteins spectra are dominated by backbone conformation signals, whereas lipids have more specific and defined peaks and can be more easily distinguished by RS. The resulting CLS fitting scores reported in Table 2 described the relative contribution of each standard molecule to PC1 and PC2 loadings, thus their contribution to the observed spectral differences between the three cytotypes. Figure 3C depicts the fitting coefficients in a bar graph, making apparent that SM and ganglioside (GM1) contribute to the shape of PC1 and PC2 loadings more than the other reference molecules. Similarly, PCh is the phospholipid which contributed most to fit the shape of PC1. In particular, the positive score attributed to SM after PC1 fitting demonstrated that it made a contribution to the spectrum of ASC-derived EVs, and this was further underlined by the negative SM score after PC2 fitting. On the contrary, GM1 and PCh were assigned a negative CLS fitting score in the case of PC1, suggesting their presence within the membrane of EVs from BM-MSCs and DFs rather than ASC-derived vesicles. After CLS fitting, the scores assigned to Chol, Cer, PE, and PA suggest their presence in the EVs from all three source cells, although they do not greatly contribute to the differences between EVs. It has to be noted that the considered lipids are only few of the constituents of vesicles, for this reason our results should be considered as hints for future studies aimed at verifying the exact membrane composition of vesicles.

**Table 2:** Classical least-square (CLS) fitting.

	% variance	CLS fitting scores							% Error
		Chol	Cer	SM	GM1	PCh	PE	PA	
PC1	35.1	0.13	−0.19	**0.76**	**−1.01**	**−0.99**	−0.31	0.6	2.12
PC2	22.7	0.6	0.55	−0.15	**−1.98**	0.34	0.33	0.14	1.82

CLS fitting scores obtained after fitting the reference spectra of cholesterol (Chol), ceramide (Cer), sphingomyelin (SM), monosialotetrahexosylganglioside (GM1), phosphatidylcholine (PCh), phosphatidylethanolamine (PE), and phosphatidic acid (PA) to PC1 and PC2 loadings. The reported fitting scores are a measure of the contribution of each molecule to the considered PC loadings and provide hints to explain spectral differences between EV spectra. The percentage of total variance of PC 1 and PC2 and the percentage of error obtained after CLS fitting are also reported.

### Raman spectroscopy can distinguish BM-MSC, ASC and DF EVs with 93.7% accuracy

**Figure 4:**
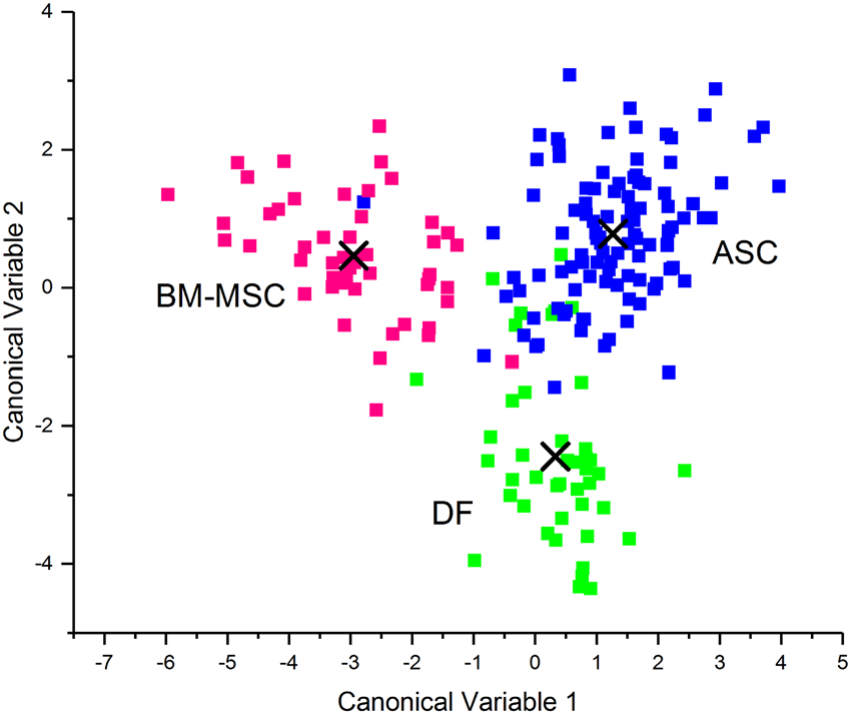
Linear discriminant analysis. The first 25 PC loadings calculated by means of PCA were used for the LDA. The scatter plot shows the LDA scores obtained for EVs from BM-MSCs (pink), ASCs (blue), and DFs (green). Each square represents a single spectrum. The crosses indicate the mean canonical observation score obtained for each group.

The first 25 PCs were used for Linear Discriminant Analysis (LDA), which made possible to verify the ability of the method to identify between-group differences by maximising the variance among classes while minimising intra-class variability. The results showed that RS clearly distinguished the biochemical fingerprints of the three groups. After leave-one-out cross-validation, the PCA-LDA model showed that the overall accuracy of the model was 93.7% and that its accuracy in distinguishing DFs from MSCs was 92% (Table 3). The LDA scatter plot (Fig. 4) revealed that the spectra of the ASC-derived EVs fell into a region that was clearly separated from those of the BM-MSC EVs. Although there was a limited overlap between the DF and ASC derived vesicles, RS distinguished their sources with a high degree of statistical confidence (Wilks’ Lambda Test, p < 0.001). Details concerning the distribution of the individual donor spectra are shown in Supplementary Figure S3.

**Table 3:** PCA-LDA confusion matrix.

		Predicted group			Total true	Sensitivity	Specificity	Accuracy
		BM-MSC	ASC	DF				
**True group**	**BM-MSC**	46	2	2	50	92%	98.7%	97.1%
	**ASC**	1	97	7	105	92.4%	91.8%	92.1%
	**DF**	1	7	35	43	81.4%	94.8%	92.1%
					198	**88.6%**	**95.1%**	**93.7%**

Confusion matrix obtained from the results of the multivariate LDA of the first 25 PCA scores after leave-one-out crossvalidation. True positives, true negatives, false positives, and false negatives were used to calculate the sensitivity, specificity and accuracy of the method.

## Discussion

The possibility of using regenerative medicine to treat diseased, damaged or aged tissues and restore organ function without side effects is one of the main challenges facing modern medical science, and so is no surprise that the discovery of the regenerative potential of EVs released by MSCs has aroused great interest. However, the main obstacle to the clinical use of vesicles is the lack of a robust and standardised method of characterising them^5^.

In this study, we investigated the biochemical fingerprints of MSC-derived vesicles originating from different tissues and compared them with those of terminally differentiated dermal fibroblasts. Our findings demonstrate the ability of RS to identify tissue-related fingerprints for vesicles released by MSCs and fibroblasts without the use of any label. This is the first time that Raman analysis has been used to provide a biochemical overview of MSC-derived EVs from a limited volume of EV suspensions.

As previously reported in relation to other types of vesicles^40, 41, 49^, our data on MSC-derived EVs confirm the ability of RS to reveal the presence of the main EV constituents in a single repeatable spectrum. Although we cannot exclude the possible presence of a limited amount of soluble factors other than vesicles in our suspension, the reproducibility of the results and the main peak attributions suggest the purity of the samples.

The main finding of this study is that RS can clearly distinguish not only vesicles from MSCs and terminally differentiated fibroblasts, but also vesicles of MSCs from bone marrow and adipose tissue. Although there are protein markers that define a stem cell phenotype exist, a straightforward distinction between bone-marrow and adipose-tissue MSCs, based on biological and functional features, is still difficult to be obtained^50^. The ability of RS to highlight unique, tissue-specific features of vesicles should therefore assist scientists working with stem cells. Even if assessing the possible correlation between biochemistry and function goes beyond the scope of this study, the biochemical variations observed provide suggestions for further investigations into the functional differences of EVs from multiple MSC types and sources for which there is still not a definite marker^51, 52^.

Analysis of the spectra of MSC- and DF-derived EVs revealed that lipids made a substantial contribution to the Raman signals, as previously reported^40, 41, 49^. The prominence of membrane constituents in determining the fingerprints of vesicles is in line with the growing body of evidence demonstrating that lipids play a crucial role in the formation of EVs^1^ and the fulfilment of their signalling functions^42^. It is known that a number of specific lipids are typically associated with lipid rafts and enriched in vesicles that inherit the plasma membrane composition of their cell of origin. In particular, cholesterol and sphingolipids are preferentially included in EV membranes and may be involved in the formation of vesicles and in their stability in the extracellular environment^42^. There is also evidence that lipids are involved in BM-MSC responses to a strongly pro-inflammatory environment^53^. Furthermore, it is possible that direct membrane interactions between vesicles and recipient cells is one of the mechanisms of action of EVs, as has already been demonstrated in the case of whole cells^2^.

On the basis of CLS fitting results, we hypothesised that gangliosides, phosphatidylcholine and sphingomyelin directly contributed to the main spectral differences between the considered EVs. Our data are in agreement with those of a recent proteomic and lipidomic study demonstrating how sphingomyelins, ceramides, cholesterol and phosphatidylcoline were enriched in the exosomes of BM-MSCs in comparison with other cell types^54^, but there is still a lack of data concerning the membrane composition of ASC-derived exosomes. Our observation that GM1 also contributes to the recorded spectra of BM-MSC vesicles is in line with the reported functional role of gangliosides in regulating the proliferation and neuronal differentiation of MSCs^55, 56^. Similarly, it is known that ceramides and ceramide-containing lipids are involved in many of the pathways mediating immune responses, and that they modulate the adipogenic differentiation of MSCs^57^. Despite the reported functional significance of PA in the biogenesis and release of exosomes, our data did not reveal any significant difference of PA content in the EVs derived from the three cell types, as has also been noticed by Haraszti *et al*.^54^ Further lipidomic studies are needed to verify the exact membrane composition of MSC-derived vesicles and to establish the role of lipid species in mediating vesicle function.

It is important to mention one limitation of our study related to the sex mismatch of our MSC donors. It is known that MSC activity and recipient responses are influenced by the sex of both donor and recipient because of circulating hormones^58^, but, to the best of our knowledge, no specific study has been published concerning sex-related variations in the function of EVs derived from cultured cells. Studies evaluating the efficacy of MSC-derived EVs *in vitro* and *in vivo* rarely refer to the sex of the donors, but this is very useful and should be always clearly indicated together with donor age^59^. Future in-depth analyses of larger donor cohorts should evaluate age and sex-related differences in EV function and chemical composition.

In conclusion, our findings demonstrate that RS can determine the chemical content of EVs in a label- and sample processing- free manner. The proposed method can be immediately transferred into laboratory practice as it allows the bulk characterisation of vesicle suspensions before their use *in vitro* or *in vivo*. As independent MSC-derived EV preparations can have different therapeutic potentials, the overall characterisation of vesicles offered by Raman spectroscopy might become a pivotal quality check for comparing data coming from different experiments or research labs, and thus hasten the clinical application of stem cell-derived products.

## Methods

All of the relevant experimental data have been submitted to the EV-TRACK knowledgebase (EV-TRACK ID: EV170012)^60^.

### Cell cultures

Human BM-MSCs were isolated from the residual bone marrow cells of healthy bone marrow (BM) transplantation donors (3 male donors, age range: 17–20 y/o) after approval by the Institutional Review Board of San Raffaele Hospital. Human ASCs and DFs were isolated from waste materials of abdominoplasty and liposuction procedures performed at IRCCS Galeazzi Orthopaedic Institute (subcutaneous adipose tissue of 4 female healthy donors - age range: 35–58 y/o - and de-epidermised dermis of 3 female healthy donors – age range: 26–46 y/o-, respectively). Tissues were collected following the procedure PQ 7.5.125 regarding waste materials to be used for research purposes, version 4 dated 22.01.2015, approved by the same institute. Written informed consent was obtained from all of the patients in accordance with the ethical principles of the Declaration of Helsinki. All of the samples were anonymised and no information or images that could lead to identification of a study participant might occur. All experiments were performed in accordance with the relevant guidelines and regulations of San Raffaele Hospital and IRCCS Galeazzi Orthopaedic Institute.

Cells were isolated following previously described protocols^61–63^. Briefly, mononuclear cells from BM aspirates were isolated by means of density gradient centrifugation (Ficoll 1.077 g/ml; Lympholyte, Cedarlane Laboratories Ltd., Burlington, Canada) and plated in non-coated 75–150 cm^2^ tissue culture flasks (BD Falcon, Franklin Lakes, NJ, USA) at a density of 160,000/cm^2^ in complete culture medium: DMEM (Euroclone, Milan, Italy) supplemented with 10% ultracentrifuged foetal bovine serum (Gibco, Life Technologies LTD, Paisley, UK), penicillin 50 U/ml, 50 µg/ml streptomycin and 2 mM L-glutamine (L-Glu, Euroclone). Cultures were maintained at 37 °C in a humidified atmosphere, containing 5% CO_2_. After 48-hour culture, non-adherent cells were removed. The ASCs were isolated from adipose tissue samples following digestion with 0.75 mg/ml type I Collagenase (250 U/mg, Worthington Biochemical Corporation, Lakewood, NJ, USA) and the filtering of the stromal vascular fraction. The DFs were obtained from de-epidermised dermis fragmented and digested with 0.1% collagenase type I. The ASCs and DFs (plating density: 10^5^ cells/cm^2^) were cultured (37 °C, 5% CO_2_) in complete culture medium. The medium was replaced every other day and, at 70–80% confluence, the cells were detached with 0.5% trypsin/0.2% EDTA, plated (BM-MSC plating density 4,000 cells/cm^2^; ASC plating density 10,000 cells/cm^2^; DF plating density 5,000 cells/cm^2^) and expanded. Once at 80–90% confluence, cells at 3^rd^–4^th^ passage were washed twice with DMEM, kept for one hour in serum-free DMEM (phenol-free DMEM supplemented with 2 mM L-glutamine, 50U/ml penicillin, 50 µg/ml streptomycin) and then cultured for 72 hours in serum-free DMEM.

### Extracellular vesicle isolation

In order to avoid the presence of RS-visible isolation reagent residues in the EV suspension, the vesicles were isolated from cell-conditioned medium (CM) by means of differential centrifugation, as previously described^43^. Briefly, after 72 hours of starvation, the medium conditioned from approximately 6 × 10^6^ cells was centrifuged at 800 g for 10 min to remove non-adherent cells and then at 2,500 g for 15 min to remove potential apoptotic bodies. CM was then ultracentrifuged for 70 min at 100,000 g (L7–65; Rotor 55.2 Ti; Beckman Coulter, Brea, CA, USA) at 4 °C, and the pellet was re**-**suspended in sterile saline solution and ultracentrifuged again. The collected EV suspension (approximately 500 µl) was kept at 4 °C before making Raman and TEM analyses, and then frozen.

### Western blotting

Immunoblotting was performed to characterise the EVs as suggested by ISEV minimal experimental requirements^44^. The EV pellets were re**-**suspended in SDS sample buffer with protease inhibitors^64^. Electrophoresis was performed under reducing conditions, and then proteins were transferred to nitrocellulose membrane. The antigens were probed with anti-flotillin-1 (BD Transduction Laboratories™, San Jose, CA, USA), anti-CD63 and anti-CD9 (System Biosciences, Palo Alto, CA, USA), and anti-calnexin (endoplasmic reticulum protein used as negative control, clone C5C9, Cell Signaling Technology, Danvers, MA, USA). As secondary antibodies goat anti-mouse (Thermo Fisher Scientific, Waltham, MA, USA) and goat anti-rabbit (System Biosciences) conjugated with HRP were used. Cell lysates were considered as the control for the specificity and working conditions of the considered antibodies.

### Transmission electron microscopy and size measurement

For the TEM visualisation of EVs, 5 µl of purified exosomes were absorbed on Formvar carbon-coated grids for 10 min. The drops were then blotted with filter paper and negatively stained with 2% uranyl acetate (5 μl) in aqueous suspension for 10 min. Excess of uranyl was removed by touching the grid to a filter paper. The grids were dried at room temperature and examined with a transmission electron microscope (Leo 812AB, Zeiss, Oberkochen, Germany) at 80 kV. The TEM images obtained in order to verify EV ultrastructure were used to assess vesicles’ size using the particle analysis tool of ImageJ software (National Institutes of Health, Bethesda, MD, USA). At least 30 measurements per sample were done.

### Raman spectroscopy

Freshly isolated EVs were analysed by means of Raman microspectroscopy (LabRAM Aramis, Horiba Jobin Yvon S.A.S, Lille, France) equipped with a diode-pumped solid-state laser operating at 532 nm and a Peltier-cooled CCD detector. 5–10 µl drops of EV suspension were deposited on a calcium fluoride slide and allowed to air dry. All of the measurement**s** were performed with 50× objective (NA 0.75, Olympus, Tokyo, Japan), 1800 grooves/mm diffraction grating, 400 µm entrance slit, and confocal mode (600 µm pinhole) in the spectral ranges 500–1800 cm^−1^ and 2600–3200 cm^−1^. Accumulation times were 2 × 10 s per spectrum.

The Raman shift was calibrated automatically using LabSpec 6 software (Horiba) using zero order line and Si line of a Si reference sample. In order to capture the spectra randomly, maps of about 150 µm^2^ (with lateral steps of 20–30 µm) were acquired in the centre and at the borders of the air-dried drops. Before analysing the data, a two-class hierarchical clustering analysis (HCA) of the Raman maps was made in order to distinguish the spectra relating to vesicles from those related to background. At least 10 independent replicates of the Raman spectra were obtained for every donor of the different cell types.

### Data analysis

Baseline correction was made using LabSpec 6 processing tool by fitting all spectra with a sixth order polynomial curve in order to remove autofluorescence and background before unit vector normalisation. Post-acquisition calibration was carried out on normalised spectra, in order to compensate for possible thermal drifts.

Principal component analysis (PCA) of the normalised and aligned spectra was made in order to reduce the dimension of the data and describe their major trends. The provided principal components (PCs) represent differences in the spectra of vesicles from the three cytotypes and therefore in their chemical composition. The first 25 PC scores were used in a supervised classification model, linear discriminant analysis (LDA), in order to discriminate and classify the data by maximizing the variance between groups. Data reduction by PCA before LDA was essential because LDA requires that the number of variables is smaller than the number of observations. The smallest number of PC scores was selected for the LDA to prevent data overfitting. A decreased number of PCs reduced the accuracy of the method in distinguishing the EVs, whereas an increased number did not improve the classification, but progressively decreased accuracy. Leave-one-out cross-validation was used to test the classification sensitivity, specificity, and accuracy of the LDA model. One-way ANOVA was performed on PC scores to verify that the means of each group were significantly different, despite within-group variance.

Data manipulations and statistical analysis were performed using Origin2017 (v. 9.4, OriginLab, Northampton, MA, USA).

### CLS fitting

Reference molecules of some of the major known constituents of EV membrane were used to investigate the lipid content of vesicles. Cholesterol (Chol), ceramide (N-stearoyl-D-erythro-sphingosine; Cer), sphingomyelin (SM), phosphatidylcholine (16:0/22:6; PCh), L-α-phosphatidylethanolamine (PE), phosphatidic acid (PA), and monosialotetrahexosylganglioside (GM1) were purchased from Avanti Polar Lipids (Alabaster, AL, USA) and used to acquire reference spectra using the same acquisition settings as those used for the EV analysis.

Labspec 6 was used for the Classical Least-Squares (CLS) fitting of the PC1 and PC2 spectra, which allows to calculate the contribution of the reference chemicals to the PC spectra by evaluating any similarities. The resulting coefficients described the relationships between the PC spectra and the reference molecules.

### Data availability

The datasets generated and analysed during the current study are available from the corresponding author on reasonable request.

## Electronic supplementary material

Supplementary Information
