# Supplementary material for: Raman spectroscopy uncovers biochemical tissue-related features of extracellular vesicles from mesenchymal stromal cells
Source: Sci Rep. 2017 Aug 29;7:9820. doi: 10.1038/s41598-017-10448-1 (PMC5575260; doi:10.1038/s41598-017-10448-1)
Supplement: Supplementary file 1 — Supplementary Information [file 41598_2017_10448_MOESM1_ESM.pdf]

Supplementary Information for:

Raman spectroscopy uncovers biochemical tissue-related features of extracellular vesicles from mesenchymal stromal cells

Alice Gualerzi[1,*], Stefania Niada[2,3], Chiara Giannasi[2,3], Silvia Picciolini[1,4], Carlo Morasso[1], Renzo Vanna[1], Valeria Rossella[5], Massimo Masserini[4], Marzia Bedoni[1], Fabio Ciceri[6], Maria Ester Bernardo[5], Anna Teresa Brini[2,3], Furio Gramatica[1]

[1] Laboratory of Nanomedicine and Clinical Biophotonics, IRCCS Fondazione Don Carlo Gnocchi ONLUS, Milano, Italy
[2] Dipartimento di Scienze Biomediche, Chirurgiche ed Odontoiatriche, Università degli Studi di Milano, Milano, Italy
[3] Laboratorio di Applicazioni Biotecnologiche, IRCCS Istituto Ortopedico Galeazzi, Milano, Italy
[4] Nanomedicine Center NANOMIB, School of Medicine and Surgery, University of Milano-Bicocca, Monza, Italy
[5] San Raffaele Telethon Institute for Gene Therapy (SR-TIGET), Pediatric Immunohematology, San Raffaele Scientific Institute, Milano, Italy
[6] Hematology and Bone Marrow Transplantation Unit, San Raffaele Scientific Institute, Milano, Italy

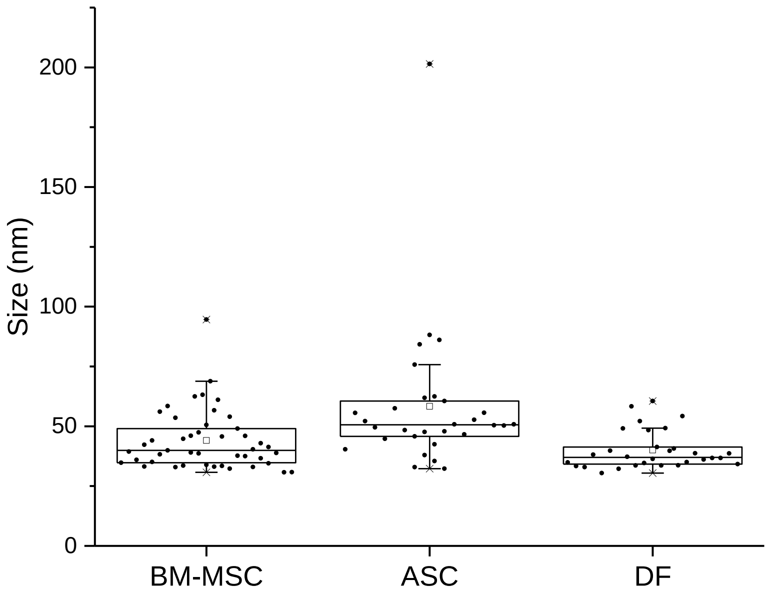

Supplementary Figure S1. Size measurements. The box plot shows the size of EVs as they were measured on TEM images by Image J. BM-MSC: 45 measurements, mean size: 46.9 nm ± 12.9 nm ; ASC: 30 measurements, mean size: 60.8 nm ± 31.2 nm; DF: 30 measurements, mean size 40 nm ± 8 nm. The size of all of the analysed vesicles ranged from 30.5 nm to 201.5 nm. The size ranges demonstrate that the considered vesicles belong to exosome and microvesicle categories.

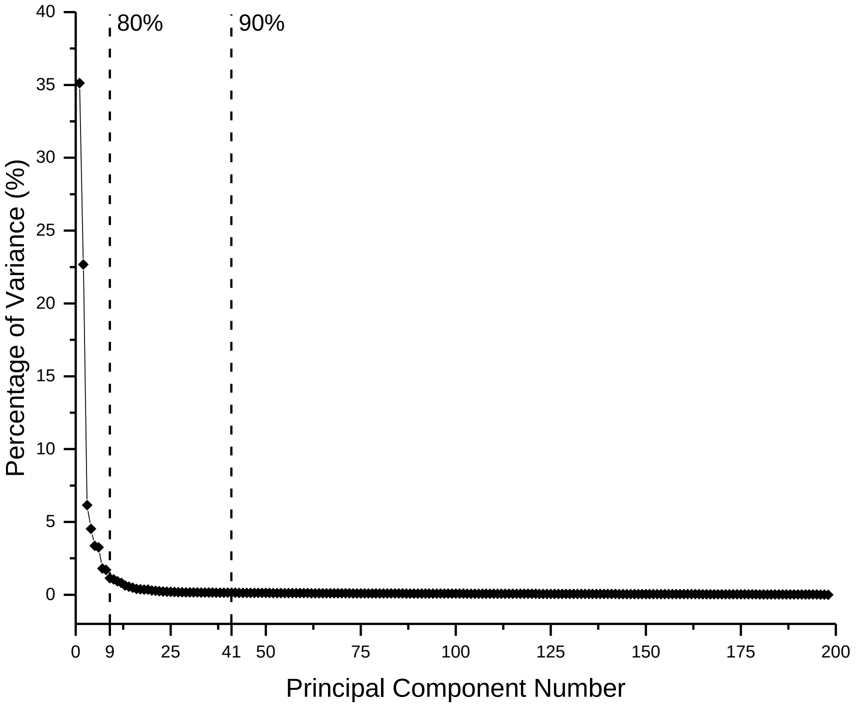

Supplementary Figure S2. Principal Component Analysis (PCA). The scree plot shows the percentage of variance of all of the Principal Components (PCs) deriving from the PCA of Raman spectra. As shown, the cumulative percentage thresholds of 80% and 90% are reached respectively with PC9 and PC41.

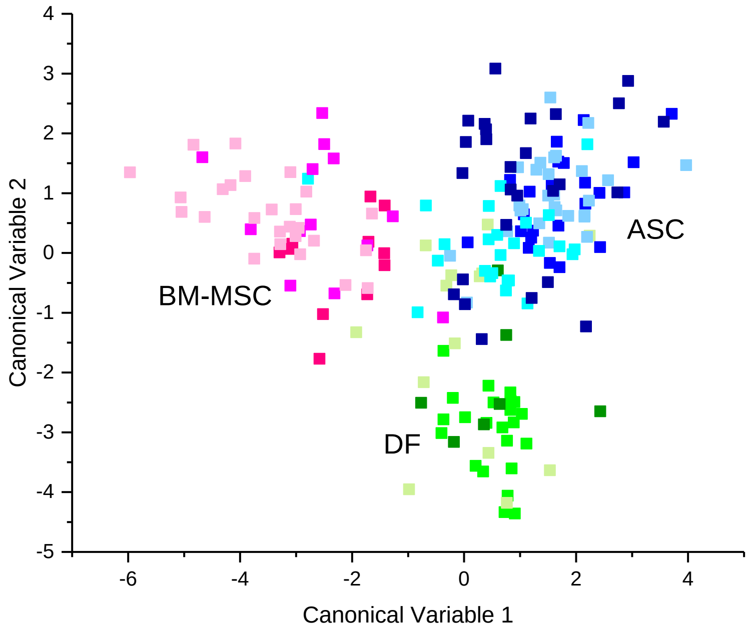

Supplementary Figure S3: Linear discriminant analysis (LDA) with donor details. The first 25 PC loadings calculated by means of PCA were used for LDA. The scatter plot shows the LDA scores obtained for EVs from BM-MSCs (pink), ASCs (blue), and DFs (green). The contribution of single donors within every cell group is highlighted with different colour shades, one for each donor.

Principal Component	Percentage of Variance (%)	Cumulative (%)
**1**	35.13	35.13
**2**	22.67	57.8
**3**	6.15	63.95
**4**	4.53	68.48
**5**	3.36	71.84
**6**	3.25	75.09
**7**	1.8	76.89
**8**	1.71	78.6
**9**	1.13	79.73
**10**	1.06	80.79
**11**	0.93	81.72
**12**	0.81	82.53
**13**	0.64	83.17
**14**	0.56	83.73
**15**	0.48	84.22
**16**	0.4	84.62
**17**	0.38	85
**18**	0.36	85.36
**19**	0.35	85.71
**20**	0.29	86
**21**	0.27	86.27
**22**	0.25	86.52
**23**	0.22	86.74
**24**	0.21	86.94
**25**	0.19	87.14

Supplementary Table S1. Principal Component Analysis (PCA). Percentage of variance for the first 25 Principal Components (PCs) resulting after PCA of EV-derived Raman spectra. The 25 PCs were used to perform the multivariate analysis LDA.

One-way ANOVA after PCA-LDA multivariate analysis					
	**R-Square**	**F Value**	**df**	**df2**	**Prob>F**
**PC1**	0.3412	50.4932	2	195	0
**PC2**	0.1535	17.6738	2	195	< 0.0001

Supplementary Table S2. One-way Analysis of Variance. Statistical analysis of between group variance of PC1 and PC2 scores. The null hypothesis assumed that there was no significant difference among the groups. The alternative hypothesis assumed that there was a significant difference among the groups. As the p value associated with the F (Prob>F) is smaller than 0.05, the null hypothesis was rejected. These results demonstrated that the mean of the groups were not equal for both PC1 and PC2, despite within-group variance.
